# Polyvinyl Chloride Nanoparticles Affect Cell Membrane Integrity by Disturbing the Properties of the Multicomponent Lipid Bilayer in *Arabidopsis thaliana*

**DOI:** 10.3390/molecules27185906

**Published:** 2022-09-11

**Authors:** Mingyang Li, Yuan Zhang, Changyuan Li, Jinxing Lin, Xiaojuan Li

**Affiliations:** 1 Key Laboratory of Genetics and Breeding in Forest Trees and Ornamental Plants, Ministry of Education, College of Biological Sciences and Biotechnology, Beijing Forestry University, Beijing 100083, China; 2 Beijing Advanced Innovation Center for Tree Breeding by Molecular Design, Beijing Forestry University, Beijing 100083, China

**Keywords:** NPs, molecular dynamics (MD) simulations, plant development, cell membrane integrity

## Abstract

The ubiquitous presence of nanoplastics (NPs) in natural ecosystems is a serious concern, as NPs are believed to threaten every life form on Earth. Micro- and nanoplastics enter living systems through multiple channels. Cell membranes function as the first barrier of entry to NPs, thus playing an important biological role. However, in-depth studies on the interactions of NPs with cell membranes have not been performed, and effective theoretical models of the underlying molecular details and physicochemical behaviors are lacking. In the present study, we investigated the uptake of polyvinyl chloride (PVC) nanoparticles by *Arabidopsis thaliana* root cells, which leads to cell membrane leakage and damage to membrane integrity. We performed all-atom molecular dynamics simulations to determine the effects of PVC NPs on the properties of the multicomponent lipid bilayer. These simulations revealed that PVCs easily permeate into model lipid membranes, resulting in significant changes to the membrane, including reduced density and changes in fluidity and membrane thickness. Our exploration of the interaction mechanisms between NPs and the cell membrane provided valuable insights into the effects of NPs on membrane structure and integrity.

## 1. Introduction

Plastics are synthetic materials composed of long chains of molecules such as carbon atoms, hydrogen, nitrogen, oxygen, and sulfur in the form of polymers. Plastics are grouped into four categories based on particle size: macroplastics (>25 mm), mesoplastics (5–25 mm), microplastics (MPs) (0.1–5 mm), and nanoplastics (NPs) (<100 nm) [1]. Nanoplastics (NPs) are typically defined as particles with a diameter between 0.1 and 100 nm, regardless of their morphological characteristics [2,3]. Due to their extraordinarily small size, NPs usually show very interesting properties, such as colloidal, bioactivity, and electric properties, which substantially differ from those of bulk materials [4]. Given the multifaceted nature of NPs, understanding their interactions with the plasma membrane (PM) is a major concern for environmental safety and human health.

The PM functions as the first barrier to the entrance of NPs into cells, thereby playing an important role in cellular interactions with and internalization of the NPs [5]. Therefore, the role of the PM in the effects of NPs on cells has attracted increasing attention worldwide. Ample evidence has shown that small microplastic particles, especially NPs, can disperse throughout various organs, passing through different biological barriers and thereby accumulating in and affecting these organs [6,7]. Because of their nanoscale size, NPs can easily enter cells and translocate across cells, tissues, and organs, thus causing greater harm than larger plastic particles [8]. Smaller polystyrene (PS) MPs can enter the cells of the green alga *Chlamydomonas reinhardtii* and become embedded in the cell membranes [9]; larger (micrometer-sized) plastic particles can also enter the interiors of cells but probably via endocytosis-like mechanisms [10,11]. Zheng et al. determined that different kinds of MPs, including PS, polyvinyl chloride (PVC), and polyethylene (PE) MPs, cause varying levels of damage to the cell membranes of freshwater algae *Microcystis aeruginosa* [12]. The principal mechanism by which cationic nanoparticles cause cytotoxicity is membrane rupture, which greatly damages the extracellular matrix and leads to the production of reactive oxygen species [13]. However, this process does not result in cell death or loss of cell membrane integrity [14]. Therefore, understanding the underlying mechanisms involved in the cellular uptake of NPs is crucial for assessing the fate of these particles and their toxicity.

Several studies have investigated the effects of exposure to MPs and NPs on plants in different ecosystems, with findings ranging from neutral effects to overt phytotoxicity [3,15,16,17]. Notably, the effects of MPs and engineered nanomaterials on plant growth vary depending on the plant species, the particle structure, size, chemical composition, and the exposure time [18,19,20]. Plant roots take up nanoparticles through the apoplast, across cell membranes by endocytosis, and through the endodermis into root vascular tissue via the symplast [21,22]. Moreover, Giorgetti et al. identified PS NPs in the nuclei of onions (*Allium cepa*), suggesting that small NPs might also cross the nuclear membrane and impair chromatin structure and activity [23]. It seems safe to assume that the smaller the particle size, the greater the cytotoxicity. Several studies have investigated the uptake, accumulation, and cytotoxicity of micro-nano plastics in plants. However, detailed cellular interaction and early responses are still very challenging.

Most studies of the interactions between MPs and NPs and plasma membranes performed to date have focused on subsequent phenomena following treatment with plastic particles. However, elucidating the initial interactions between plastic particles and lipid membranes is crucial to understanding the physical basis of membrane damage caused by NPs in vivo. However, the considerable complexities of membranes and the difficulty of examining NPs smaller than the diffraction limit make systematic research based on standard experimental methods quite challenging. Due to the rapid development of computational approaches, studies have increasingly focused on predicting interactions between macromolecules and cell membranes via molecular dynamics (MD) simulations [24,25]. Wang et al. used MD simulations to examine the effects of nano-sized PE particles on the performance of a model dipalmitoyl phosphatidylcholine membrane and determined that these nanoparticles could readily penetrate lipid membranes [8]. MD simulation is an effective tool for exploring interactions between molecules and lipid bilayer systems at atomic-level accuracy to reveal the underlying mechanisms of physiological processes that cannot be uncovered by laboratory experiments [26,27].

In this study, we combined experimental and fully atomistic MD simulation approaches to investigate the integrity and conformational dynamics of the PM. We also used MD simulation to track the changes in membrane structure and function after PVC NPs enter the mixed lipid bilayer. Elucidating the interactions between nanoparticles and lipid membranes is crucial for understanding the physical basis and in vivo processes underlying the membrane damage caused by NPs.

## 2. Results

### 2.1. Effects of NPs on Seed Germination and Plant Growth

**Figure 1:**
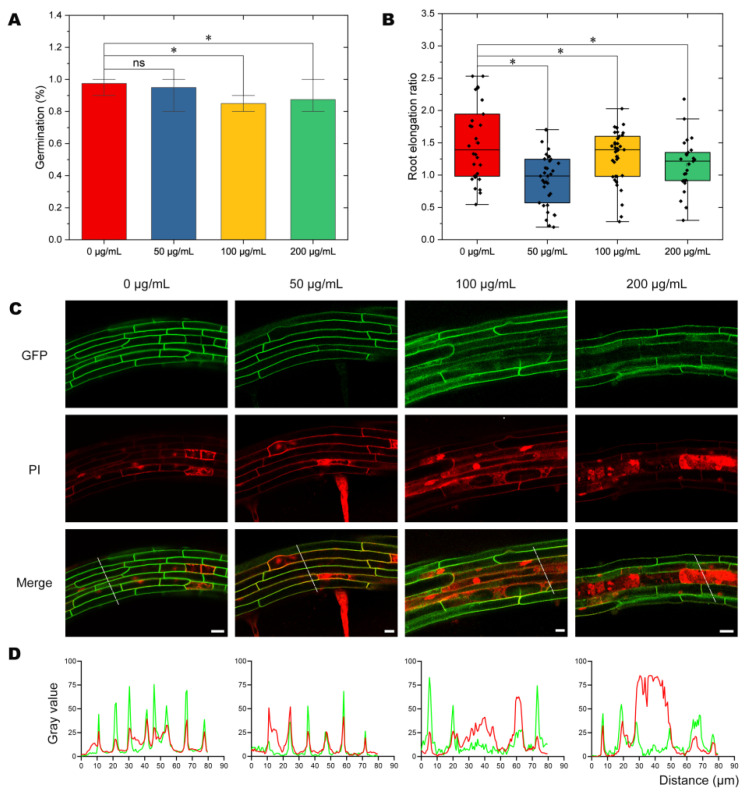
Exposure to PVC NPs alters seed germination and plant growth. (**A**) Seed germination rate after 72 h of exposure to PVC NPs. (**B**) Root elongation rate after 7 d of exposure to PVC NPs. Black dots represent the number of replicates. Seedlings of *Arabidopsis thaliana*, ecotype Columbia-0 grown on plates were analyzed in (A,B). (**C**) Integrity assay of root cortex tissues exposed to different concentrations of PVC NPs. Seedlings expressing GFP-LTi6a, which is an integral plasma membrane protein, were stained by PI. (**D**) Plot of intensity values along the white line in (**C**). The green curve represents the gray value of GFP fluorescence, and the red curve represents the gray value of PI fluorescence. Differences calculation using *t*-test were considered significant at *p* < 0.05, as indicated by *. “ns” means “no significant difference”. n ≥ 30 seedlings in **A** and **B**. Scale bars = 20 μm.

To investigate whether treatment with NPs would have negative effects on plants, we carried out plant health assessments, including germination and root elongation rate, in Arabidopsis. We analyzed the germination rate of Arabidopsis seeds following 72 h of exposure to PVC10 nanoparticles (polyvinyl chloride chains containing 10 monomers) (Figure 1). We found no statistically significant differences in germination rate between seeds treated with 0 μg/mL (controls) vs. 50 μg/mL NPs (Figure 1A). However, seeds exposed to higher doses of NPs (100 μg/mL and 200 μg/mL) showed germination delays of approximately 12.5% and 10%, respectively, compared to the 0 μg/mL controls (Figure 1A).

Root elongation was also significantly inhibited by exposure to PVC10 NPs. The growth rate between 5 and 7 d after the start of germination was lower by 7.4% in seedlings exposed to 200 μg/mL PVC10 NP treatment (Figure 1B). These results suggest that both seed germination and root growth are inhibited by long-term exposure to NPs.

### 2.2. Analysis of Cellular Integrity after NP Treatment

To investigate the effect of PVC NPs on the membrane integrity of Arabidopsis root cells, we performed a live-death assay applying propidium iodide (PI) on the roots of seedlings. PI is a widely used red, fluorescent DNA binding dye. It cannot penetrate the cell membrane of living cells, and therefore it is commonly used to detect cell membrane damages [28,29]. Damage to the cell membrane can be assessed by examining the fluorescence of PI in the intracellular compartment by confocal microscopy (Figure 1C). The PI fluorescent signals in the intracellular compartments of cells exposed to different concentrations of NPs revealed that the degree of membrane damage increased with increasing PVC treatment (Figure 1D). Notably, cells in plant cortex tissues showed greater membrane damage with increasing NP concentration (Figure 1C). These data indicate that NPs damage the integrity of the PM in a dose-dependent manner.

### 2.3. Structural and Conformational Properties of the Lipid Bilayer

**Figure 2:**
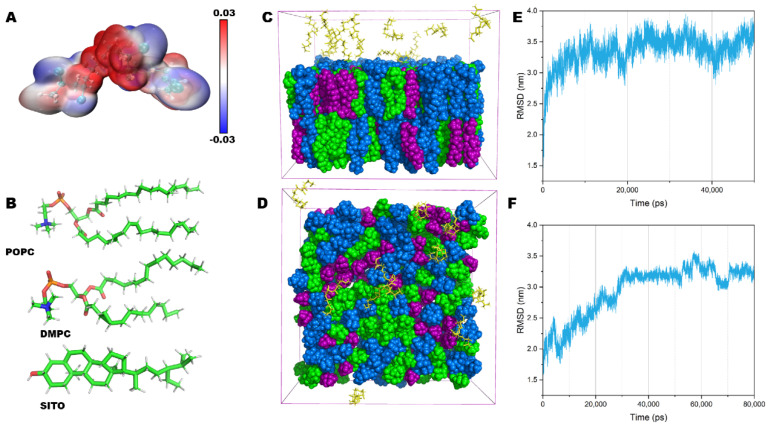
All-atom molecular models of PVC nanoparticles and the lipid membrane complex used in the MD simulations. (**A**) Chemical structure and electrostatic potential of the PVC10 monomer. The color scale bar is shown with the corresponding ESP values (a. u.) (**B**) Structures of the POPC, DMPC, and SITO lipid molecules in the model membrane. POPC (1-palmitoyl-2-oleoyl-sn-glycero-3-phosphocholine), DMPC (1,2-dimyristoyl-sn-glycero-3-phosphocholine), and SITO (sitosterol). (**C**,**D**) Lateral view (**C**) and top view (**D**) of a typical configuration of PVC10 monomers and a mixed lipid bilayer in a water box are shown in green, blue, and purple, respectively, and the PVC10 monomers are shown in orange. (**E**,**F**) Root mean square deviation (RMSD) plots for the simulation system in the absence (**E**) and presence (**F**) of PVC10 NPs.

To investigate the interaction between PVC10 (Figure 2A) NPs and the lipid bilayer in more detail, we performed all-atom MD simulations. We generated model lipid bilayers containing 256 lipid molecules using CHARMM-GUI. These simulated structures were derived from an initial model bilayer comprising a mixture of 1-palmitoyl-2-oleoyl-*sn*-glycero-3-phosphocholine (POPC), 1,2-dimyristoyl-*sn*-glycero-3-phosphocholine (DMPC), and sitosterol (SITO) (Figure 2B), at a molar ratio 38:34:28 [27] and with dimensions of 9 × 9 nm (Figure 2C,D). This membrane composition and distribution mimic a putative plant PM. All MD simulations (100-ns simulations for each replicate system) were performed with CHARMM36 all-atom force field to simulate an accurate membrane environment. At near room temperature (298 K), the root mean square deviation (RMSD) values demonstrated that after 10 ns of MD simulation, the membrane structure reached a stable balance, with the background values located at 3.5 nm (Figure 2E). Figure 2F shows a plot of RMSD vs. 80 ns MD simulation for a bilayer membrane in the presence of PVC10 NPs. Under these conditions, the membrane reached a stable state after at least 30 ns, with a decreased value of 3.1 nm (Figure 2F). Thus, the presence of the PVC10 NPs increased the equilibration time of the membrane.

### 2.4. NPs Insert into the Lipid Bilayer in Numerous Simulations

**Figure 3:**
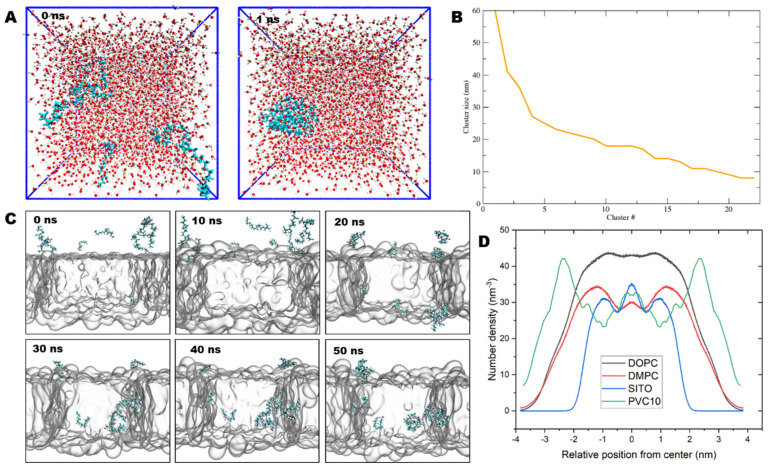
The process of PVC10 NP insertion into the lipid bilayer. (**A**) Snapshots of PVC10 monomers forming compact spheres in the water phase. (**B**) Distribution plot of NP cluster size in the water phase as a function of cluster number. (**C**) Snapshots of 50 ns MD simulations for the random and passive distribution of PVC10 NPs in the water phase above the bilayer. (**D**) Density profiles for the simulated lipid bilayer system in the presence of PVC10 NPs.

In the water system, the highly hydrophobic PVC chains rapidly formed compact aggregates (Figure 3A) in only 1 ns, with a diameter of up to 10 nm (Figure 3B). We also simulated PVC NPs in the presence of mixed model bilayers containing POPC, DMPC, and SITO mimicking the plant cell membrane. The NPs were initially placed in the aqueous phase of the system with an initial distance of 30 Å from the membrane surface. In all cases, once the PVC chain approached a sufficient gap between lipid head groups, the nanoparticles entered the carbonyl region of the bilayer on a time scale of a few nanoseconds (Figure 3C, 10 ns). Permeation into the hydrophobic core of the membrane (Figure 3C, 20–30 ns) was followed by embedding among the acyl chains of the surrounding phospholipids on a time scale of 1−10 ns. Notably, we observed membrane insertion of the NPs for several, but not all, of the trajectories (Figure 3C, 50 ns). PVC10 chains were confined to the centers of the lipid bilayers, as shown by the density and distance profiles of PVC10 and lipids along the *z* dimension (Figure 3D). As expected, the dynamics and behavior of PVC10 were physically different in the aqueous and lipid phases.

### 2.5. PVC Chains Alter the Homeostasis of Model Membranes

**Figure 4:**
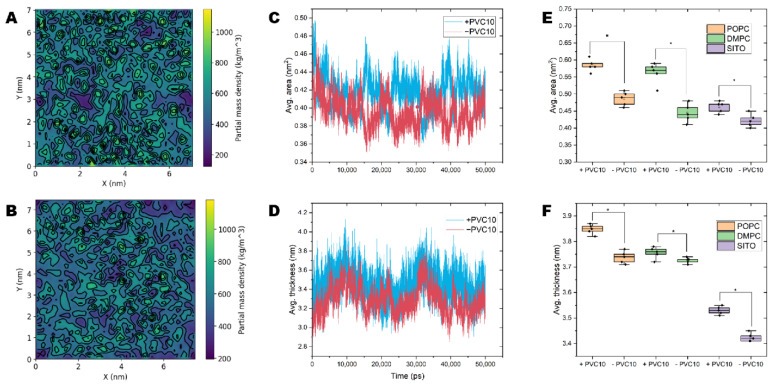
NPs alter the homeostasis of model membranes and lipids. (**A**,**B**) Partial density landscape for model membranes containing and not containing PVC10 NPs, respectively. (**C**) The average area versus time for all steps of model membranes containing (+ PVC10) and not containing (−PVC10) NPs. (**D**) The average thickness versus time for all steps of model membranes containing and not containing NPs. (**E**) The average area of individual lipids versus time in the presence (+PVC10) and absence (−PVC10) of NPs. (**F**) The average thickness of individual lipids versus time in the presence and absence of NPs. * indicates a significant difference in *p* < 0.05.

To gain further molecular insight into the specific interactions and interaction energies that drive the permeation between NPs and the membrane, we quantified the densities and areas of the lipids in the model membranes, which were defined as the average P–P or O–O (sitosterol) atomic distance in the lipid head groups (Figure 4A–D). We observed large variations in the lipid area and thickness in model membranes containing NPs (Table 1). For model membranes lacking NPs, the areas of POPC, DMPC, and SITO were 0.47, 0.46, and 0.37 nm^2^, respectively. By contrast, in the presence of NPs, the lipid area expanded, with an average value of 0.58, 0.57, and 0.45 nm^2^ for POPC, DMPC, and SITO, respectively (Table 1 and Figure 4E).

The presence of NPs in the lipid biolayer increased the area per molecule of individual lipids in the solid phase (Figure 4C,E), confirming that NPs interact with membrane lipids. Moreover, the lipid layers in membranes containing NPs were more densely packed and had a larger thickness than those in NP-free membranes (Figure 4D,F). In contrast, membranes without NPs tended to show looser packing and lower lipid bilayer thickness (Figure 4D,F).

### 2.6. NPs Affect the Lateral Organization of Multicomponent Membranes

**Figure 5:**
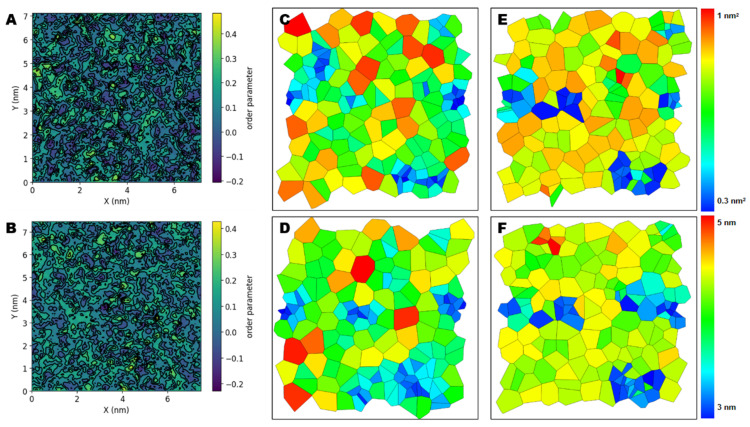
NPs alter the equilibrium properties of lipid bilayers. (**A**,**B**) Membrane order landscape for model membranes containing and not containing PVC10 NPs, respectively. (**C**,**D**) Voronoi analysis of *x*–*y* projections of the center of mass of the lipids used to obtain area per lipid values in membranes containing and not containing PVC10 NPs. (**E**,**F**) Voronoi analysis of *x*–*y* projections of the center of mass of the lipids were used to obtain the thickness per lipids in membranes containing and not containing PVC10 NPs. The colormaps were scaled differently in these images to illustrate the different values.

Whether the presence of NPs alters the homeostatic dynamics of lipids within bilayers is potentially crucial to their effects on cells. Therefore, we examined the effects of PVC chains on the properties of realistic models of biological membranes comprising different lipid species. We examined three metrics related to membrane structure: lipid density, membrane thickness, and lipid order. Compared to the neat lipid bilayers observed in the absence of NPs, we observed a change in the overall dynamic characteristics of the membrane when NPs were present. In our simulations, performed at room temperature, the multicomponent lipids formed liquid-ordered (Lo) and liquid-disordered (Ld) domains with well-defined compositions (Figure 5A). By contrast, in systems with NPs (Figure 5B), the membrane average order parameters were significantly lower (0.213) than the value in systems without NPs (0.291, statistical error < 0.05).

Area per lipid is an average value that does not provide information about the distribution of lipid molecules. To examine possible changes in lipid distribution, we performed a Voronoi analysis on the *x-y* plane to obtain the probability distributions for area and thickness per lipid. The areas of Voronoi cells describe the area and thickness distribution of the lipids’ centers of mass. In the ordered state in the absence of NPs, many lipids occupied a small area per lipid (Figure 5C–D, shown in blue). Notably, the highly disordered nature of membranes containing NPs led to increasing membrane thickness (Figure 5E–F, shown in red).

### 2.7. The Interaction Energy between PVC10 and the Lipid Bilayer

**Figure 6:**
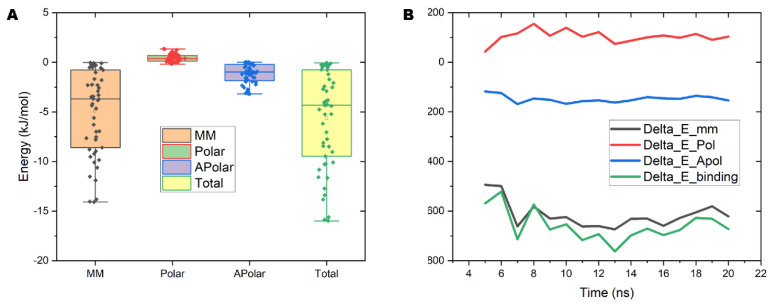
Decomposition and distribution of the binding free energy of PVC10 permeation in the lipid bilayer. (**A**) Decomposition of the binding free energy of PVC10-membrane complexes using g_mmpbsa. Sample sizes are shown as individual dots within each box (MM, molecular mechanics energy, Polar, polar solvation energy; apolar, nonpolar solvation energy; Total, total binding energy). (**B**) Distribution of the binding free energy of the PVC10-membrane system versus time.

To quantify the mechanical properties and interactions between the NPs and lipid bilayers, we calculated the interaction energy between these components (Figure 6A). We performed a per-atom decomposition of the binding free energy to identify the residues with the greatest contributions to self-association free energy. The binding energy is decomposed into individual components, including molecular mechanics (MM), polar solvation energy (polar), and nonpolar solvation energy (apolar). The average values of total binding energy for PVC10 and lipid membrane is −4.8 kJ/mol, which indicates that PVC10 has easy binding interaction and insertion to the lipid membrane (Figure 6A). The binding free energy curves for PVC10 molecules crossing the lipid bilayer indicate that the value of binding free energy reached a minimum when molecules were at the center of the lipid bilayer (Figure 6B, green line). During permeation from the water phase to the head of the lipid layer, an apparent barrier (115 kJ/mol) should be overcome at 7000 ps to 8000 ps (Figure 6B). Based on the free energy profile, the PVC10 and lipid bilayer interaction appears to be more favorable for the permeation of NPs, with a negative value of −523 kJ/mol. At the end of the 50-ns MD simulation, 7 of the 10 PA molecules were stably and fully inserted into the middle of the lipid bilayer. These results indicated that PVC10 NPs spontaneously translocated from the aqueous phase to the center of the bilayer.

### 2.8. NPs Induce Pore Formation in the Lipid Bilayer

**Figure 7:**
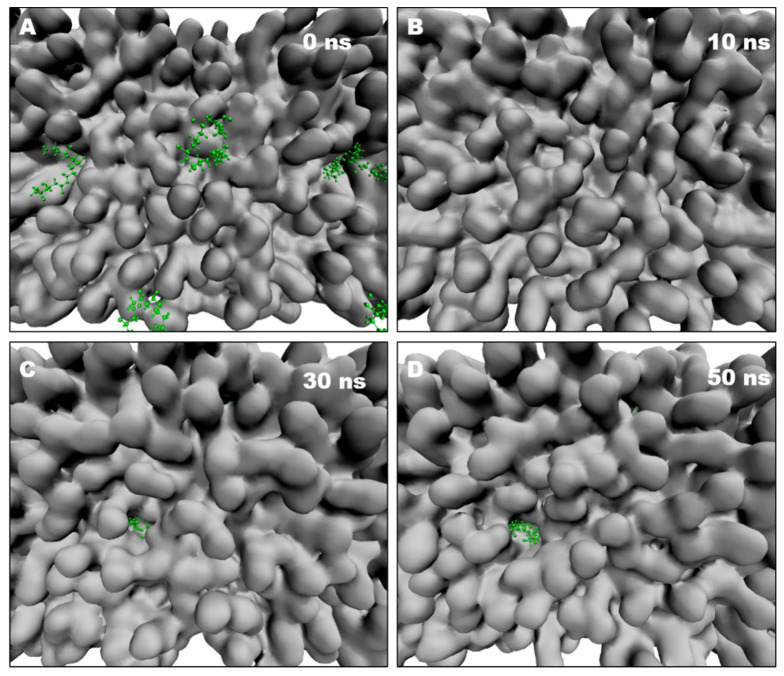
Membrane pore formation is induced by NPs. (**A**) Top view of a lipid bilayer with 10 PVC10 monomers. (**B**) Intact lipid bilayer before pore formation (10 ns from the start of the simulation). (**C**) Pore formation in the hydrophobic lipid tails induced by NPs (starting from the intact lipid bilayer shown in A, at 30 ns). (**D**) Expansion to a larger pore (~1 nm in diameter) with lipids around the pore (at 50 ns). The lipids composition is in silver and the PVC10 molecules are in green.

As NP treatment leads to membrane leakage in Arabidopsis root cells, we analyzed the membrane–water interface of the lipid bilayer. At 10 ns after the start of an MD simulation, there was no apparent change in the surface of the membrane in the presence of NPs in the aqueous phase (Figure 7A,B). After 30 ns, NPs had contacted and entered the membrane, leading to disordered sorting and the formation of pores on the surface of the model bilayer (Figure 7C). Subsequently, the PVC10 molecules tended to disperse, occupying more space inside the membrane, which led to the expansion of membrane pores (Figure 7D). In addition, following pore formation, a few free water molecules outside the lipid bilayer entered the interior of this structure. At the same time, pore formation might also lead to the leakage and outflow of substances from the cell, as revealed in membrane leakage experiments performed by PI staining (Figure 1C).

## 3. Discussion

Plastics are versatile materials with a wide range of properties, chemical compositions, and applications that have become essential to many aspects of human life during the past half century. Although MPs and NPs are constantly being released, and persist in the ecosystem, only very recently were these contaminants identified. Since then, an increasing number of studies have demonstrated the adverse effects of these particles on plant and animal communities. MPs were found to successfully enter *Chlamydomonas reinhardtii* cells and become embedded in cell membranes, whereas larger PS MPs could not enter the interiors of these cells [9]. Notably, NPs < 100 nm in diameter were found to penetrate cell membranes more easily than larger particles, becoming distributed throughout the organism [30,31,32]. In the present study, we determined that NPs had mixed effects on Arabidopsis seed germination and root elongation: the highest doses of NPs reduced seed germination, whereas the lower doses caused increased root elongation. Treatment with 100-nm PS nanoparticles also increased root elongation in wheat (*Triticum aestivum*) [33]. These data suggest that plants have a complex, dose-dependent response to NPs, with different sizes and concentrations having different effects.

MPs and NPs enter the cell by passing through biological barriers, thereby affecting the structure and function of the cell. Cell membranes act as the first barriers to the entrance of plastic particles into cells and have important biological effects, which are inevitably influenced by MPs during this process. Cell membranes are among the most important barriers in cells, as they selectively control the entry and exit of substances via diffusion, infiltration, and active transport to maintain normal cellular metabolism. In general, polar or charged molecules that cannot pass through the hydrophobic PM are internalized through a form of active transport known as endocytosis. Endocytosis of NPs has been widely observed in animals, perhaps representing a route for the cellular uptake of NPs [34]. The interaction between NPs and the PM might also be related to the accumulation of these particles in intracellular compartments in the root epidermis due to the small size these particles [17,23]. Regardless of the method employed, there are always limits on the detection precision of NPs and of their dynamic interactions with PM, mainly due to the particles’ extraordinarily small size.

How NPs enter a cell is a key factor determining the molecular changes that they trigger and their toxicity to the cell. The interactions between NPs and biological membranes are difficult to experimentally study in living systems at the molecular level, primarily due to the limited resolution of conventional optical techniques (~200 nm). MD simulation, which can quantify the relevant properties of particles and provide detailed atomic information, is a powerful tool for exploring the interactions and effects of NPs on the PM [8,27]. Several well-known MD simulation packages can be used to perform dynamics simulations in a single or multiple graphical processing units (GPUs) with performance that exceeds that of even the most powerful conventional central processing unit (CPU)-based supercomputers [26,35,36]. The rapid advances in supercomputing techniques and algorithms make it possible to capture large molecular systems at long temporal and spatial scales approaching true experimental conditions [37,38].

Here, through all-atom MD simulations of lipid bilayers combined with PCV10 NPs, we detected a decrease in the lipid order and increase in membrane thickness of a model membrane comprising three lipid components, which is consistent with previous results obtained using a dipalmitoyl phosphatidylcholine bilayer [8]. NPs can also noticeably increase the area per lipid compared to those of membrane systems without NPs. Finally, the permeation of NPs into membranes appears to be a spontaneous process driven by the minimization of the binding free energy of NP binding with lipids. Therefore, we conclude that exposure of the nonpolar hydrophobic core of NPs to the polar water phase drives the automatic insertion of NPs into the PM.

## 4. Materials and Methods

### 4.1. Plant Materials

*Arabidopsis thaliana* ecotype Col-0 and GFP-Lti6a [39] seeds were surface sterilized in 70% ethanol for 2 min and 5% (*w*/*v*) NaClO for 15 min. After being washed three times with sterile distilled water, the seeds were vertically grown on half-strength Murashige and Skoog (1/2 MS) medium solidified with 1% agar, pH 5.8, at 22 °C under a 16-h/8-h light/dark cycle.

### 4.2. Plant Cell Labeling and Treatment with PVC NPs

The PVC NPs were purchase from Shanghai Fengtai Plastic & Chemical Co., Ltd. Seedlings were treated by adding PVC nanoparticles to solid 1/2 MS medium (The culture medium was configured with 1/2 MS 1.1 g/500 mL, agar 4 g/500 mL, sucrose 5 g/500 mL), nanoplastics at a final concentration of 50, 100, or 200 μg/mL. To calculate the relative elongation ratio of roots, the difference in the root length from 5 to 7 d old were divided by the length of the lengths of 5 d old. For PI staining, 5 d old Arabidopsis seedlings were incubated in 1/2 liquid MS medium containing 5 mg/L propidium iodide (PI, Sigma-Aldrich, St. Louis, MO, USA) for 10 min and visualized under a confocal microscope. A total of 20 seedlings were analyzed in analysis of difference significance.

### 4.3. Confocal Microscopy Detection

The PI-stained roots were observed under a laser-scanning confocal microscope (Leica SP8, Germany) equipped with a filter (excitation filter, 450–490 nm; barrier filter, 520 nm). Data were collected from five plants per treatment, and each treatment was replicated three times. Images were processed using ImageJ software (National Institutes of Health, Bethesda, MD, USA, Version 1.53r). Available from https://imagej.nih.gov/ij/, accessed on 8 September 2022. 10 September 2022.

### 4.4. Atomistic Molecular Dynamics Simulation

Simulated mixed lipid bilayers were generated using the CHARMM-GUI Membrane Builder [40]. The main simulation system for each model contained 256 lipids evenly distributed in two leaflets with neutralizing ions and was fully hydrated using TIP3P water. All atomistic molecular dynamics simulations were performed using GROMACS 2019.3 [41], with CHARMM36 (CGenFF) force field parameters [42]. The simulations were performed at a temperature of 298 K using a Berendsen thermostat with τp = 0.1 ps. Periodic boundary conditions and the particle-mesh Ewald algorithm [43] were used to account for long-range electrostatic effects. Bond lengths were constrained using the LINCS method. Coordinates were saved every 2 ps for subsequent analysis. Energy minimization was performed, followed by equilibration (position restraint for 50 ps) using NPT ensembles with a semi-isotropic coupling constant τP = 1.0 ps and compressibility = 4.5 × 10^−5^ bar^−1^. All MD simulations were conducted using five independent sets for systems in the presence and absence of PVC10 NPs.

### 4.5. Molecular Dynamics Simulation Analysis

**Mass density:** Membrane thickness, and lipid ordering plots were calculated using analysis software packages developed by the Luca group in IBCP (CNRS), France. All landscapes were calculated using g_thickness, g_ordercg, and g_mydensity software [44], which is freely available at http://perso.ibcp.fr/luca.monticelli, accessed on 8 September 2022. The lipid order parameter, defined as *p* = 1/2 < 3 cos^2^θ − 1 >, where θ is the angle between the bond vector and the membrane normal, was calculated for all bonds in the lipid acyl chains and then averaged within each voxel over the ensemble of bonds and over time. The binding free energy was calculated using the molecular mechanics Poisson Boltzmann surface area (MM-PBSA) method [45] with the g_mmpbsa code. Voronoi diagrams of the individual area per lipid and lipid thickness values in the mixed bilayers were obtained using the APL@Voro program [46]. The MD trajectories were visualized, and all snapshots in this article were created using VMD software [47].

## 5. Conclusions

Taken together, our findings indicate that PVC NPs damage plants and the plasma membranes of cells. The resulting structural and dynamic changes in the bilayer alter vital functions of the cell membrane, potentially resulting in cell death. In addition, our MD simulations of the interactions between PVC NPs and a model membrane indicated that the PVC chain could easily enter the lipid bilayer. The permeation of NPs altered several key physical properties of the model membrane, resulting in changes in lipid area, lipid density, lipid order, and membrane thickness. In addition, the presence of NPs in the lipid bilayer tended to induce the formation of membrane pores, which might represent the key factor causing membrane leakage and damage. Further characterization of the behavior of NPs in the PM will require techniques with higher spatial resolution, such as single-particle tracking [48,49] and super-resolution microscopy (PALM and STED) [50,51]. Nonetheless, our in-depth studies of the interactions between NPs and the cell membrane shed light on the physical basis of the membrane damage caused by NPs and should facilitate experimental studies in this field.

## Figures and Tables

**Table 1:** Effect of PVC10 NPs on membrane properties.

	Membrane in the Presence of NPs					
	Upside Layer			Downside Layer		
Tested Lipids	Avg. Area (nm^2^)	Avg. Thickness (nm)	Sum. Area (nm^2^)	Avg. Area (nm^2^)	Avg. Thickness (nm)	Sum. Area (nm^2^)
POPC	0.58	3.87	21.49	0.56	3.83	21.08
DMPC	0.57	3.78	19.18	0.59	3.81	19.92
SITO	0.45	3.53	11.98	0.44	3.52	12.71
PVC10	0.16	1.84	7.87	0.42	3.88	9.58
	**Membrane Not Containing NPs**					
POPC	0.47	3.77	17.37	0.45	3.84	16.72
DMPC	0. 46	3.75	15.27	0.49	3.95	16.39
SITO	0.37	3.45	10.69	0.36	3.51	10.49

## Data Availability

Not applicable.

## Sample Availability

Samples of the *Arabidopsis thaliana*, ecotype Columbia-0, GFP-LTi6a are available from the authors.
